# Development of Highly Soluble Anthraquinone Dichroic Dyes and Their Application to Three-Layer Guest-Host Liquid Crystal Displays

**DOI:** 10.3390/ma2041636

**Published:** 2009-10-23

**Authors:** Hiroki Iwanaga

**Affiliations:** Corporate Research & Development Center, Toshiba Corporation, 1 Komukai-Toshiba-cho, Saiwai-ku, Kawasaki 212-8582, Japan; E-Mail: hiroki.iwanaga@toshiba.co.jp; Tel.: +81-44-549-2174; Fax: +81-44-549-2387

**Keywords:** anthraquinone dichroic dyes, coumarin dye, solubility, three-layer GH-LCDs, fluorinated liquid crystals

## Abstract

Therelationships between molecular structures and properties of anthraquinone dichroic dyes were studied and dyes with large solubilities and dichroic ratios were developed. The yellow anthraquinone dye behaves as a quencher of the coumarin dye, and the mixture has a large absorption coefficient without fluorescence. These technologies can enlarge the color reproduction area of three-layer guest-host liquid crystal displays (GH-LCDs) 1.6-fold. The performances of the prototype reflective three-layer GH-LCDs are as follows: the white state luminous reflectance is 43% and the contrast is 5.3, indicating that they are promising candidates for portable information systems with full-color images.

## 1. Introduction

**Figure 1:**
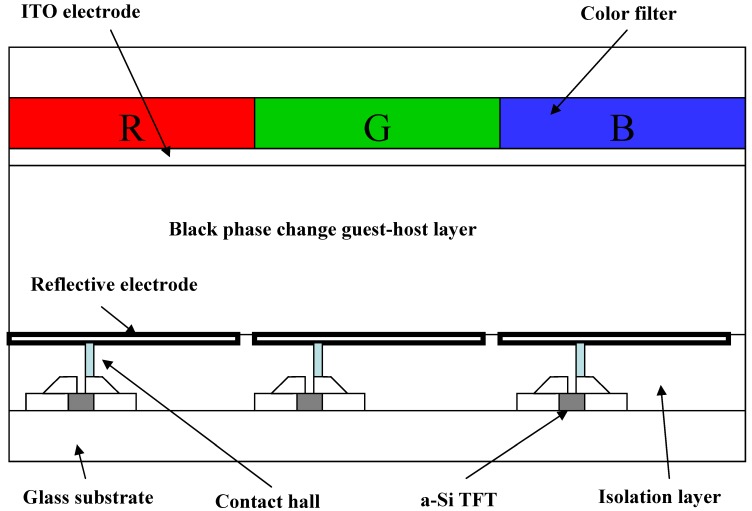
The structure of the reflective phase change GH-LCD.

Reflective liquid crystal displays (LCDs) are suitable for portable information systems because of their low power consumption. The reflective guest-host mode liquid crystal displays (GH-LCDs), wherein dichroic dyes are dissolved in liquid crystals were developed and attracted much attention [1]. However, the absorption of a polarizing plate is very high (~50%) and brightness is diminished drastically. In order to improve display performances, it is necessary to select a LCD mode operating without a polarizing plate. Some LCD modes without a polarizing plate were reported [2,3,4,5,6,7,8,9,10]. Among them, thin film transistor (TFT) driven reflective phase change GH-LCDs have a relatively wide viewing angle, high reflectance and are very promising display modes for future applications [9,10]. The schematic structure of reflective phase change GH-LCD with color filter consisting of red, green and blue is shown in Figure 1.

In the case of the RGB color filter GH-LCDs using black GH liquid crystals, color images are created by color filters and the black GH liquid crystal layer has a light-bulb function. The contrast is high and color reproduction areas are large. However, since the color filters always absorb the light, light utilization efficiency is limited. This means that images obtained from displays of this type are insufficient in some cases, with the insufficiency being particularly marked when the displays are viewed in dark places. On the other hand, bright images were obtained by using color filters consisting of yellow, magenta and cyan [9]. However, the color reproduction areas are considered to be drastically decreased compared with RGB color filter GH-LCDs.

**Figure 2:**
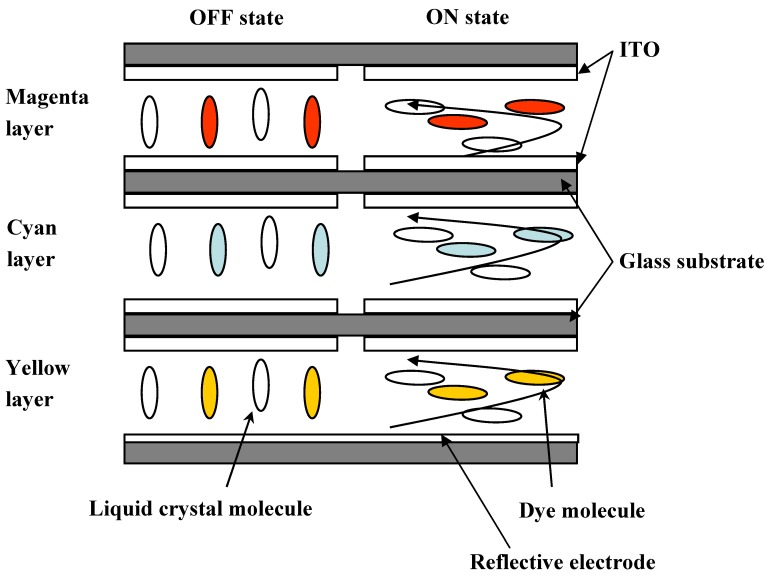
The schematic structure of the three-layer reflective GH-LCD using negative liquid crystals.

The concept of the three-layer GH-LCDs, which require no color filters and a polarizing plate, has been reported [11]. The reflective three-layer GH-LCDs, consisting of yellow, magenta and cyan guest-host layers, are most desirable because a bright full-color image is expected to be realized [12,13,14,15,16]. The schematic structure is shown in Figure 2.

Each GH layer is driven by TFT individually, and black, white, yellow, cyan, magenta, red, green, blue and their intermediate colors are created by all pixels followed by constructing full-color images. The properties of dichroic dyes are important for the realization of high performances of the three-layer GH-LCDs. In particular, absorption spectra (color), solubilities and dichroic ratios in liquid crystals are the dominant properties. 

Dichroic dyes have been developed mainly to make black dyes for monochrome displays by mixing [17]. The spectrum requirements of dyes for the three-layer GH-LCDs are much higher than those for monochrome displays, and are even higher than those for photography from the viewpoint of color contrast [14]. Azo dyes have been actively investigated [6], but they are unsuitable for three-layer GH-LCDs because of their excessively wide absorption spectra that reduce their color reproduction area. On the other hand, anthraquinone dyes not only satisfy the spectrum requirement, but also have wide color selectivity and excellent photostability [18,19,20,21,22,23]. 

In particular, the anthraquinone dichroic dyes with the phenylthio groups or the anilino groups at α positions are intramolecular charge transfer complexes and reported to be dissolved in cyanobiphenyl liquid crystals to some extent [22,23]. However, the cyanobiphenyl liquid crystals can’t be applied to the thin film transistor liquid crystal displays (TFT-LCDs) because they require high resistance values. 

**Figure 3:**
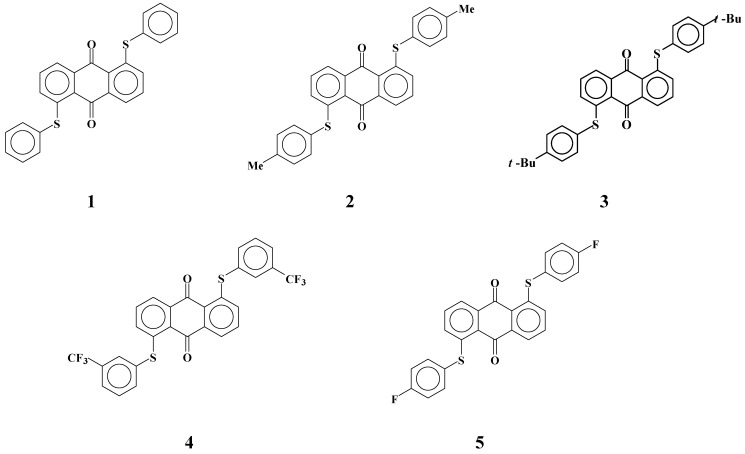
The molecular structures of yellow anthraquinone dichroic dyes with phenylthio groups used in this study.

Fluorinated liquid crystals with high resistance were developed for TFT-LCDs [24,25]. However, the solubilities of yellow anthraquinone dyes **1**-**5** with phenylthio groups (Figure 3) in fluorinated liquid crystals were much smaller than those in cyanobiphenyl liquid crystals [26]. This problem is most serious for yellow anthraquinone dyes having a small absorption coefficient, and therefore larger concentrations of dyes are required. Moreover, GH-LCDs require dichroic dyes with large solubility even at low temperatures to protect dyes from recrystallization in the liquid crystal cell.

The purpose of this study is to enlarge the performances of the three-layer GH-LCDs by realizing highly soluble anthraquinone dichroic dyes in fluorinated liquid crystals even at low temperatures. The ultimate aim is to develop portable information systems with full-color images.

## 2. Molecular Structures and Properties of Anthraquinone Dichroic Dyes 

### 2.1. The fundamental molecular structures of dichroic dyes for the three-layer GH-LCDs

The preferable wavelength ranges of yellow, magenta and cyan colors of the three-layer GH-LCDs are as follows from the analogy of photographic dyes: yellow 425-470 nm, magenta 525-560 nm, and cyan 630-670 nm. The Pariser-Parr-Pople configuration interaction (PPP-CI) quantum chemical calculation method [27] was used to select the molecular structures of dyes [14]. The PPP calculation deals only with π-electrons. Nevertheless, the results are sufficiently reliable because π-electrons determine most of the optical and electrical properties. Various properties of large molecules can be calculated without exact knowledge of the coordinates of atoms in dye molecules.

**Figure 4:**
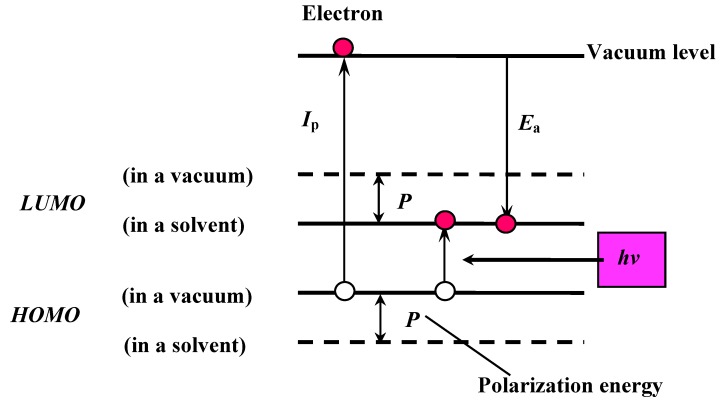
The correlation of molecular energy levels between in a vacuum and in a solvent.

The PPP method is semi-empirical. The determination of the calculation parameters is the most important step to obtain reliable and quantitative results. The correlation of molecular energy level between in a vacuum and in a solvent is shown in Figure 4. 

The optical properties of the studied dyes are those in liquid crystals, not those in a vacuum. However, quantum calculations deal with molecules in a vacuum. In liquid crystal solvent, ionic species are stabilized by the polarization energy (*P*). The polarization energy is generated by reorientation of atoms or molecules and by localization of electric charges. Therefore, in liquid crystals, obtained ionization potential values (*I*_p_) decrease, whereas obtained electron affinity values (*E*_a_) increase. In the calculation, this corresponds to the fact that HOMO and LUMO energy levels become substantially shallower and deeper, respectively. Excited states obtained by light absorption have polar structures and are generally stabilized by the polarization energy. Therefore, estimation of *P* is important in the PPP method. 

In order to estimate the polarization energy (*P*), *I*_p_ parameters in the PPP program were modified so that these parameters for donor heteroatoms were decreased and those for acceptor heteroatoms were increased. In the calculation, an *I*_p_ increment corresponds to an *E*_a_ increment.

Calculated suitable fundamental molecular structures of yellow, magenta and cyan anthraquinone dyes for the three-layer GH-LCDs are shown in Table 1 [14].

Yellow and magenta anthraquinone dyes **A**~**C** were synthesized according to reference 22. Cyan dyes **D** and **E** were newly synthesized [28]. The absorption coefficient (*ε*) of yellow anthraquinone dye **A** is small, and is consistent with the calculated small oscillator strength (*f* = 0.37).

**Figure 5:**
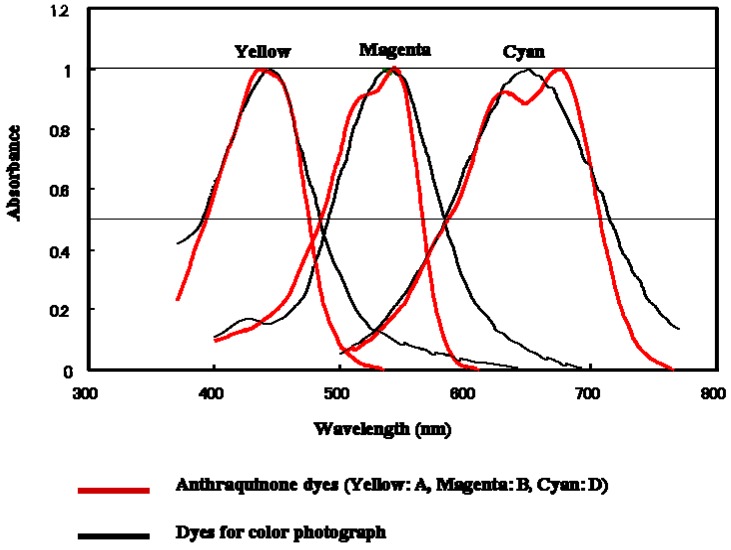
The comparison of absorption spectra of anthraquinone dyes for the three-layer GH-LCDs and photographic dyes.

Figure 5 indicates absorption spectra in dilute ethyl acetate solutions. The spectrum features were suitable for the three-layer GH-LCDs. In concentrated liquid crystal solutions, λ_obs_ increased by 10~15 nm. The half-width values of anthraquinone dyes were narrower than those of photographic dyes resulting in purer color image.

The simple PPP-CI method was found to be extremely useful in investigation related to guest dyes. Yellow, magenta and cyan dyes could be optimized from the viewpoint of absorption properties.

**Table 1:** Extracted molecular structures of yellow, magenta and cyan dyes for the three-layer GH-LCDs from the viewpoint of absorption spectra. 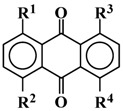

	Color	R^1^	R^2^	R^3^	R^4^	λ_obs_(nm)^1)^	λ_cal_(nm)^2)^	*f* ^3)^
A	Yellow	SPh	H	H	SPh	435	433	0.37
B	Magenta	SPh	SPh	SPh	SPh	524	513	0.52
C	Magenta	SPh	SPh	OH	OH	537	541	0.63
D	Cyan	SPh	SPh	NH(p-*n*-Bu-Ph)	NH(p-*n*-Bu-Ph)	646	664	0.78
E	Cyan	SPh	SPh	NH(*n*-Bu)	NH(*n*-Bu)	638	598	0.71
^1)^ Maximum wavelength of absorption spectra (measured in ethyl acetate at room temperature).								
^2)^ Calculated maximum wavelength.								
^3)^ Oscillator strength.								

### 2.2. Development of yellow anthraquinone dyes with two different phenylthio groups at 1 and 5 positions for the three-layer GH-LCDs

**Figure 6:**
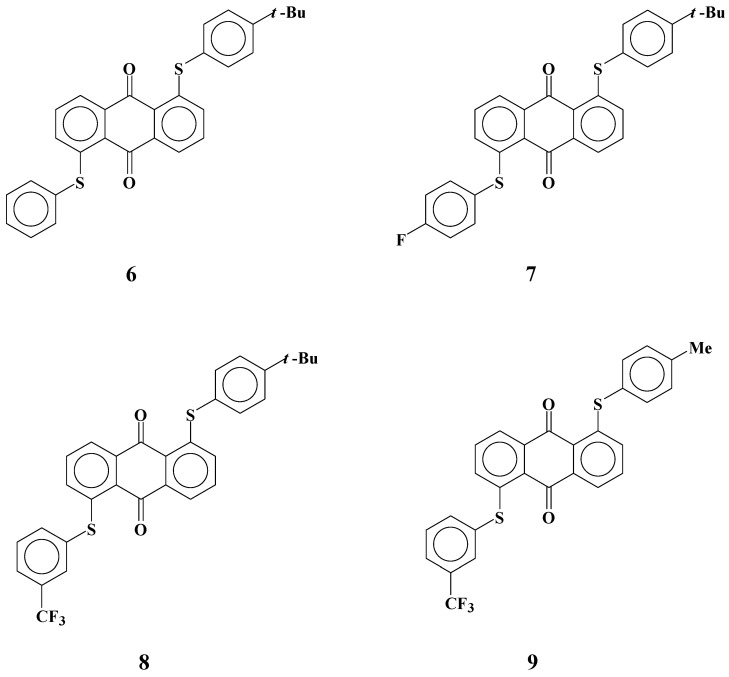
The molecular structures of yellow anthraquinone dyes with two different phenylthio groups at 1 and 5 positions.

Several yellow anthraquinone dyes **6**-**9** with phenylthio groups at the 1 and 5 positions were synthesized (Figure 6) [26,29]. Two different phenylthio groups were introduced to form asymmetric molecular structures to be dissolved in fluorinated liquid crystals. Dye **6** has the simplest asymmetric molecular structure. The molecular structure of dye **7** includes a fluorine atom at the *para* position of a non-substituted phenylthio group to clarify the effect of introduction of fluorine atom on solubility.

The molecular structure of dye **8** is the same as that of dye **6**, except that a trifluoromethyl group is added at the *meta* position of a non-substituted phenylthio group, and dye **9** is the same as dye **8** except that it has a methyl group at the *para* position instead of a *t*-butyl group. The solubilities of these dyes in fluorinated liquid crystals at room temperature and low temperatures and their dichroic ratios are shown in Table 2. The fluorinated liquid crystal material used in this study was LIXON 5052 XX produced by Chisso Corp. Solubilities of some dichroic dyes in several fluorinated liquid crystal materials were measured and LIXON 5052 XX was selected as the standard in view of its preferable results. The properties of LIXON 5052XX are as follows: dielectric constant (horizontal) is 7.0; dielectric constant (vertical) is 3.3; and nematic and liquid phase change temperature is 376.8 K.

**Table 2:** The relationships between molecular structures of yellow anthraquinone dyes and their solubilities and dichroic ratios.

Dye	Solubility (wt%)^1)^		Dichroic ratio
	r. t.	low temp. ^2)^	
**6**	4.0	1.7	1.7
**7**	3.1	2.1	2.1
**8**	5.8	3.5	3.5
**9**	0.66	0.38	0.38
^1)^ Measured in fluorinated liquid crystals (LIXON 5052XX).			
^2)^ 267-268 K.			

As shown in Table 2, unlike symmetric dyes, asymmetric anthraquinone dyes **6**-**8** are soluble to some extent in fluorinated liquid crystals. In particular, the solubility of dye **8** at room temperature reached 5.8 wt%, and remained at a high level (3.5 wt%), even at low temperatures. To realize bright and clear colors, solubility above 3 wt% is required for yellow anthraquinone dyes with relatively small absorption coefficients. No previous yellow anthraquinone dichroic dyes were reported to satisfy this level of solubility. The solubility of dye **6** was large at room temperature and decreases little even at low temperatures. However, dye **8** with a trifluoromethyl group at the *meta* position was more soluble than dye **6** without a trifluoromethyl group. In general, it is expected that trifluoromethyl group will induce a large dipole moment in dye structures. Therefore, the interaction between dye molecules will increase, resulting in low solubility. However, to compare dyes **6** and **8**, introduction of a trifluoromethyl group enlarges the solubilities. Another important feature of these asymmetric dyes is that the solubilities at low temperatures were very large, whereas solubilities usually drastically decrease at low temperatures compared with those at room temperature. Two possibilities are considered. One is that the introduction of a trifluoromethyl group at a *meta* position induces greater molecular asymmetry and interactions between dye molecules decreased. The other is that the introduction of a local polar structure that causes an electrostatic interaction with liquid crystal molecules is effective for enlarging the solubility. Dichroic ratios of dyes **6**~**9** are large enough (10~11) for application to GH-LCDs with efficient contrast. 

### 2.3. Yellow anthraquinone dyes with a different phenylthio group restricted in their flexibility

**Figure 7:**
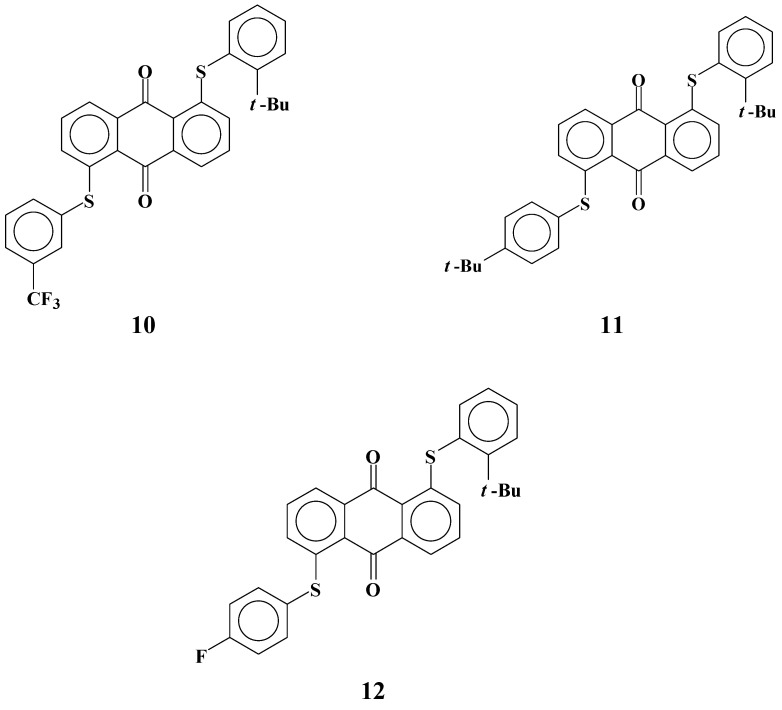
The molecular structures of yellow anthraquinone dyes with an *ortho*-substituted phenylthio group.

When electrostatic interactions between dye **8** and liquid crystal molecules increase the solubility, the flexibility of phenylthio groups has a predominant role in forming a suitable conformation enabling strong interactions of dyes and liquid crystals. Yellow anthraquinone dyes **10**-**12** with phenylthio groups at the *ortho* positions that restrict the flexibility of substituents were synthesized to clarify this thesis [29] (Figure 7). The solubilities of these dyes in fluorinated liquid crystals at room temperature and low temperatures and their dichroic ratios are shown in Table 3.

**Table 3:** The relationships between molecular structures of yellow anthraquinone dyes and their solubilities and dichroic ratios.

Dye	Solubility (wt%)^1)^		Dichroic ratio
	r. t.	Low temp.^2)^	
**10**	**1.0**	**0.5**	**7.2**
**11**	**1.8**	**1.6**	**7.9**
**12**	**1.4**	**1.3**	**7.8**
^1)^ Measured in fluorinated liquid crystals (LIXON 5052 XX).			
^2)^ 267 - 268 K.			

Solubilities of *ortho*-substituted dye **10** are much smaller than those of *para*-substituted dye **8**, despite their having the same molecular structure except for the position of the *t*-butyl group. Comparison of dyes **7** and **12** leads to the same conclusion. These results indicate that flexibility of phenylthio substituents has a significant role in increasing the solubilities. Dichroic ratios of dyes **10** and **12** are much lower than those of dyes **8** and **7,** respectively. This drastic decrease of dichroic ratios cannot be fully explained by the smaller molecular length of dyes **10**-**12**. Non-fluorinated dye **11** was investigated to distinguish the effects of *ortho*-substitution, and the same levels of solubilities and dichroic ratios as dyes **10** and **12** were obtained.

From these results, the hypothesis of a dichroic dye in liquid crystals was established. A dichroic dye in liquid crystals forms a suitable conformation to adjust to the liquid crystal phase, which is different from the conformation in isotropic solvents. In this conformation, the solubilities and dichroic ratios increase because the interactions between the dye and liquid crystal molecules are strengthened. The dyes with limited flexibility make it hard to form a suitable conformation and have inferior properties.

### 2.4. The mixture of anthraquinone dyes with two phenylthio groups and the coumarin dye as a yellow layer of three-layered GH-LCDs [30]

**Figure 8:**
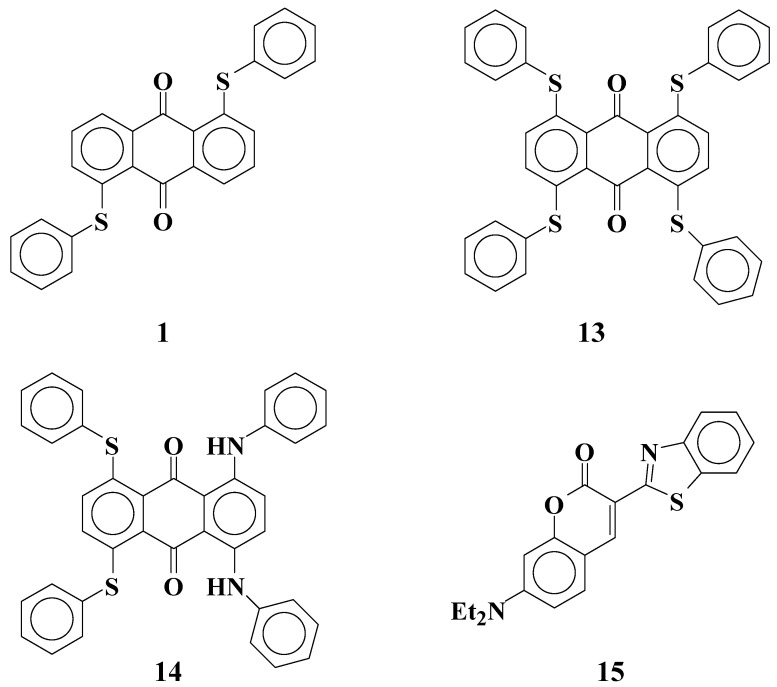
The molecular structures of calculated anthraquinone and coumarin dyes.

Table 4 lists the observed and calculated maximum absorption wavelength (λ_obsd_ and λ_calcd_, respectively), the observed half bandwidth of absorption spectra (*HW*: the width of the absorbance range when the absorbance is half of the maximum), and calculated oscillator strengths (*f*) of anthraquinone dyes and coumarin dye (Figure 8).

**Table 4:** The observed and calculated properties of anthraquinone and coumarin dyes.

Dye	λ_obsd_^1)^	λ_calcd_	*HW* (nm)	*f*
**1**	435	433	82	0.37
**13**	524	513	84	0.52
**14**	646	664	124	0.78
**15**	441	437	64	1.3
^1)^ Measured in ethyl acetate.				

**Figure 9:**
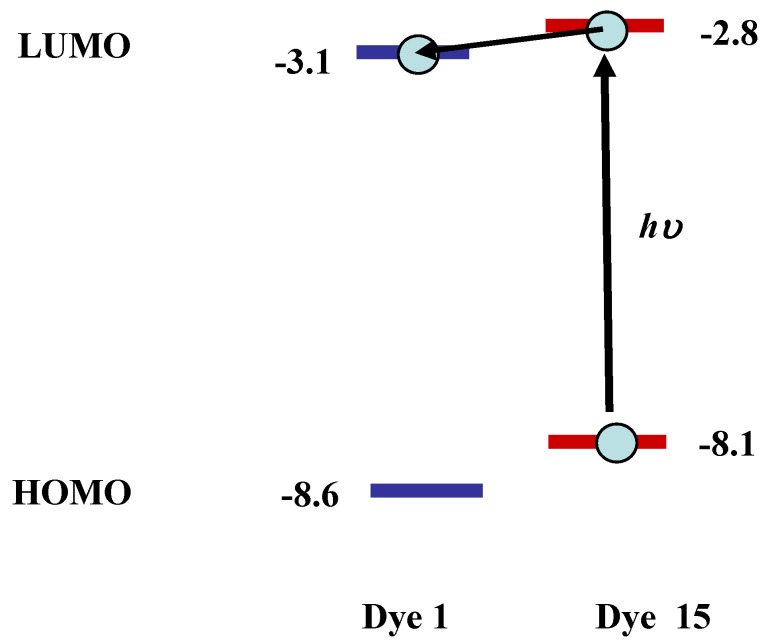
The calculated energy levels (eV) of anthraquinone dye **1** and coumarin dye **15**.

From the results listed in Table 4, the *ε* value of yellow anthraquinone dye **1** is predicted to be small, because its calculated oscillator strength (*f* = 0.37) is small. On the other hand, coumarin dye **15** is expected to have a large *ε* value (*f* = 1.30) and act as a yellow dichroic dye. However, there are problems with coumarin dye **15**, namely, its strong fluorescence adversely affects the hues it exhibit. To diminish the fluorescence of coumarin dye **15**, selection of an efficient quencher is considered to be effective. Yellow dichroic dye quencher that diminishes fluorescence of coumarin dye efficiently is the best one. The calculated HOMO and LUMO energy levels of anthraquinone dye **1** and coumarin dye **15** are shown in Figure 9. 

The lower HOMO and LUMO energy levels of anthraquinone dye **1** relative to coumarin dye **15** indicate that electron transfer will occur from coumarin dye in the excited state to anthraquinone dye. Strong fluorescence of coumarin dye is expected to be quenched through the mechanism. 

**Figure 10:**
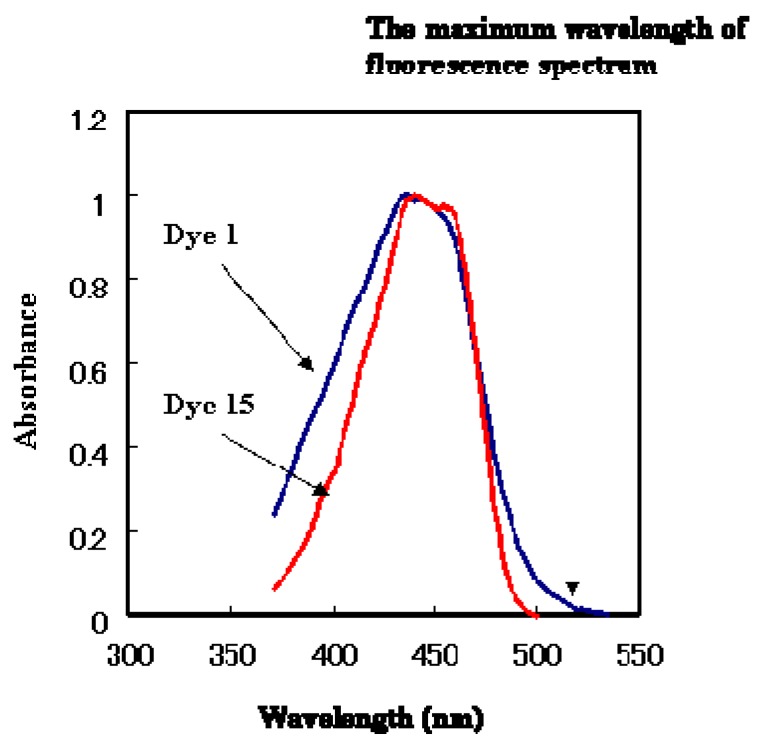
The absorption spectra of anthraquinone dye **1** and coumarin dye **15** (in ethyl acetate).

**Figure 11:**
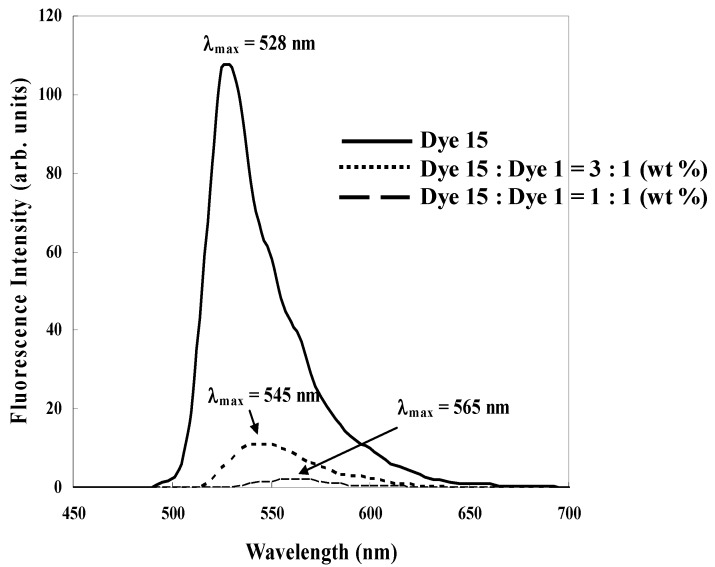
The fluorescence spectra of the mixtures of anthraquinone dye **1** and coumarin dye **15**.

There is another possible fluorescence quenching mechanism. The absorption spectra of dyes **1** and **15** are shown in Figure 10. The wider absorption tail (>500 nm) of dye **1** relative to dye **15** implies that fluorescence is quenched by energy transfer. For these two reasons, anthraquinone dye **1** is thought to behave as an efficient quencher of coumarin dye **15**. The fluorescence spectra of the mixtures of anthraquinone dye **1** and coumarin dye **15** in ethyl acetate at 297 K are shown in Figure 11.

As the concentration of anthraquinone dye **1** increases to the constant concentration of coumarin dye **15**, a rapid decrease of the fluorescence intensity was observed, as predicted. Only the ethyl acetate solution of anthraquinone dye **1** was found to have little fluorescence. Fluorescence intensities were measured in a very dilute solution. The fluorescence quenching was therefore considered to occur by energy transfer. Increasing the ratio of anthraquinone dye **1** to coumarin dye **15** causes the maximum wavelength of the fluorescence spectra to shift to a longer wavelength. This result indicates that an excitation complex of anthraquinone and coumarin dyes has been formed.

These studies show that the mixture of yellow anthraquinone dye with two phenylthio groups at the 1 and 5 positions and yellow coumarin dye gives a good yellow spectrum, weak fluorescence and large absorption coefficient, and effective for realizing GH-LCDs with high performances.

### 2.5. Magenta anthraquinone dyes with four phenylthio groups or two anilino groups

**Figure 12:**
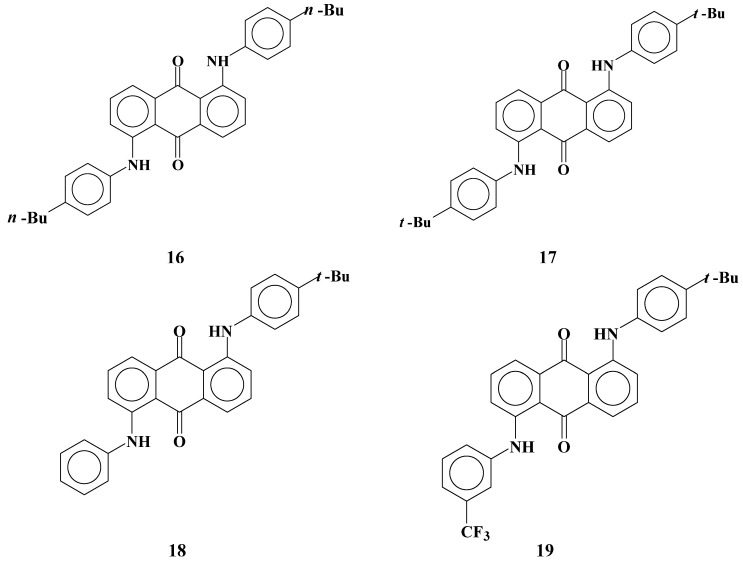
The molecular structures of magenta anthraquinone dichroic dyes with two anilino groups at the 1 and 5 positions.

Anthraquinone dyes with two anilino groups at the 1 and 5 positions are widely known magenta dichroic dyes. Solubilities and dichroic ratios of dyes **16**-**19** (Figure 12) were investigated.

**Table 5:** Solubilities and dichroic ratios of magenta anthraquinone dyes with two anilino groups at 1 and 5 positions.

Dye	Solubility (wt%)^1)^		Dichroic ratio
	r. t.	low temp.^2)^	
**16**	2.3	0.11	6.7
**17**	0.23	0.04	6.7
**18**	1.6	0.6	6.7
**19**	0.35	0.03	5.5
^1)^ Measured in fluorinated liquid crystals (LIXON 5052 XX).			
^2)^ 267 - 268 K.			

Dye **16** with flexible substituents (*n*-butyl groups) is highly soluble at room temperature. Dye **17** has the same molecular structures as dye **16** except that the substituents of the anilino groups were *t*-butyl groups instead of *n*-butyl groups, and solubilities are very low compared with those of dye **16**. Introduction of flexible groups is effective for enlarging solubilities at room temperature. However, the solubility of dye **16** decreases severely at low temperatures [26]. This result indicates that when the GH-LCDs are kept at low temperatures, dye crystallization may take place. Therefore, introduction of flexible substituents in dyes to increase solubility, which is the usual way of increasing solubility, is not suitable for molecular design of dyes used at low temperatures in large concentration. 

The asymmetric dye **18** with two different anilino groups is highly soluble at room temperature, and the solubility decreased little even at low temperatures. The asymmetric dye **19**, which has the same molecular structure as dye **18** but with trifluoromethyl groups introduced at *meta* positions of an non-substituted anilino group was found to have a relatively lower solubility. Unlike in the case of dye **8** with phenylthio groups, introduction of trifluoromethyl groups has a negative effect on increasing solubility. Moreover, dichroic ratios of dyes shown in Table 5 are much lower than those of dyes shown in Table 2, even if the substituents of phenyl groups are the same. This is considered to be due to the difference in the connection part between an anthraquinone skeleton and phenylthio or anilino groups. Flexibility of anilino groups is considered to be inferior to that of phenylthio groups for the following reasons. The length of C-N bond is smaller than that of C-S bond. Bond orders of anthraquinone dyes with phenylthio and anilino groups were calculated by the PPP-CI method (Table 6).

**Table 6:** The bond orders of C-N and C-S bonds calculated by PPP-CI methods. 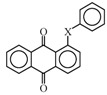

X	Bond order	
	Ph (C)-X	Anthraquinone (C)-X
**S**	0.32	0.39
**NH**	0.44	0.54

The bond orders of C-N bonds were found to be larger than those of C-S bonds. Stronger double bonding properties of C-N bonds are considered to restrict the flexibility of anilino groups. Inner molecular hydrogen bond also restricts the flexibility of anilino groups. The anilino group signal in the NMR spectra of dye **16** is shifted to lower magnetic fields from the original chemical shift and is very broad [^1^H-NMR (400 MHz, CDCl_3_): δ_H_ 11.3 (2H, s, br, NH)]. These results means that intramolecular hydrogen bonds are formed in dye **16** and rotation of anilino groups are restricted. 

**Figure 13:**
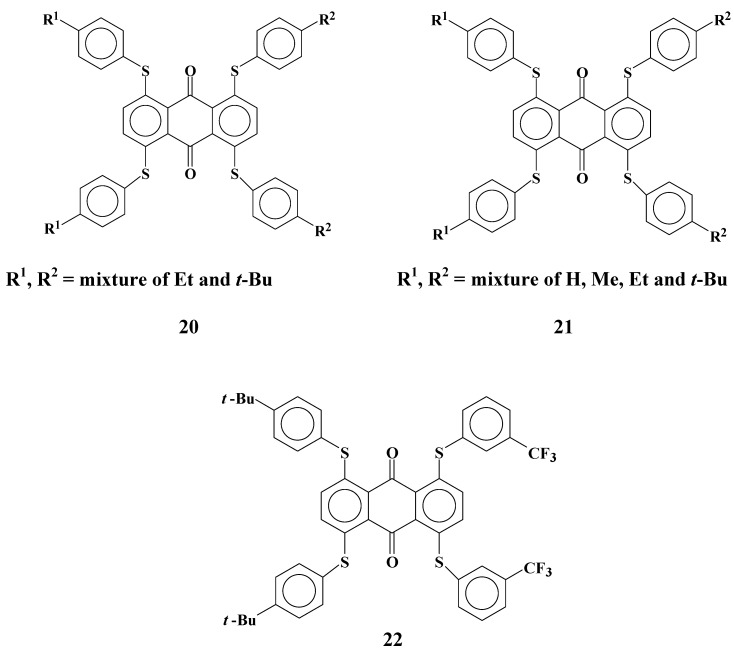
The molecular structures of magenta dichroic dyes with phenylthio groups at the 1, 4, 5 and 8 positions.

Anthraquinone dyes with phenylthio groups at the 1, 4, 5 and 8 positions are selected for magenta dichroic dyes for the three-layer GH-LCDs as described in section 2.1. The synthesized molecular structures of magenta dyes **20**-**22** are shown in Figure 13. Solubilities and dichroic ratios of these dyes are listed in Table 7.

Dyes **20** and **21** are mixtures of several dyes, but solubilities at low temperatures are not very large. On the other hand, as in the case of yellow anthraquinone dye **8**, solubilities of dye **22** are very large even at low temperatures. These results mean that the combination of *p*-*tert*-butylphenylthio and *m*-trifluoromethylphenylthio groups drastically increases the solubility. Moreover, dichroic ratios of dyes **20**-**22** are preferable for the three-layer GH-LCDs with large contrast.

**Table 7:** Solubilities and dichroic ratios of magenta anthraquinone dichroic dyes with phenylthio groups at the 1, 4, 5 and 8 positions.

Dye	Solubility (wt%)^1)^		Dichroic ratio
	r. t.	low temp.^2)^	
**20**	0.31	0.031	10
**21**	1.35	0.18	10
**22**	1.70	1.5	10
^1)^ measured in fluorinated liquid crystals (LIXON 5052 XX).			
^2)^ 267 – 268 K.			

### 2.6. Cyan anthraquinone dyes with two phenylthio groups and two anilino groups

**Figure 14:**
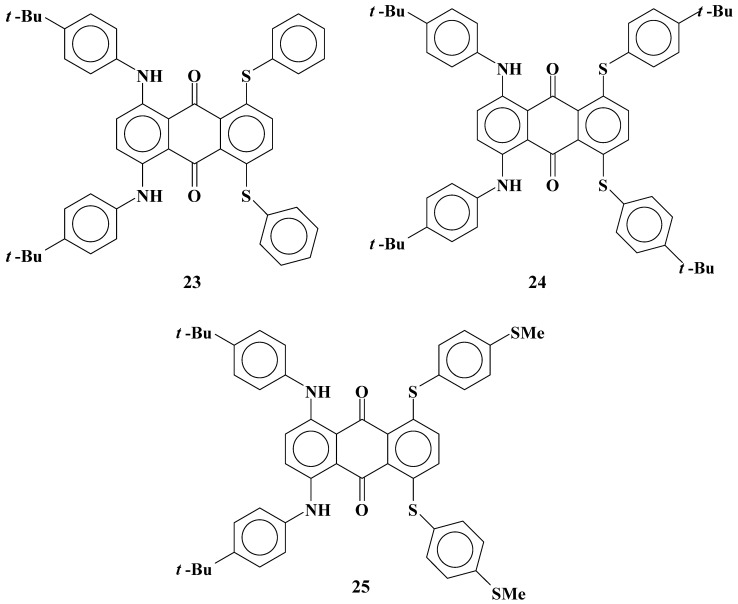
The molecular structures of cyan anthraquinone dyes with two phenylthio groups and two anilino groups.

Cyan anthraquinone dyes **23**~**25** with two phenylthio groups and two anilino groups were synthesized (Figure 14).

Solubilites and dichroic ratios of dyes **23**~**25** are shown in Table 8.

**Table 8:** Solubilities and dichroic ratios of cyan anthraquinone dichroic dyes with two phenylthio groups and two anilino groups.

Dye	Solubility (wt%) ^1)^		Dichroic ratio
	r. t.	low temp.	
**23**	0.06	0.04	5.1
**24**	0.09	0.06	5.7
**25**	0	0	Cannot be measured
^1)^ Measured in fluorinated liquid crystals (LIXON 5052 XX).			
^2)^ 267 – 268 K.			

Solubilities and dichroic ratios of anthraquinone dyes **23**~**25** are very low compared with those of dyes **20**~**22**. This marked difference from the results of anthraquinone dyes with four phenylthio groups is considered to be due to the effects of the connection parts between an anthraquinone skeleton and substituents, namely, S or NH. This result is the same as that for dye **8** with two phenylthio groups and that for dye **19** with two anilino groups.

### 2.7. Difference of the structures of solvation in liquid crystals and isotropic phase

**Figure 15:**
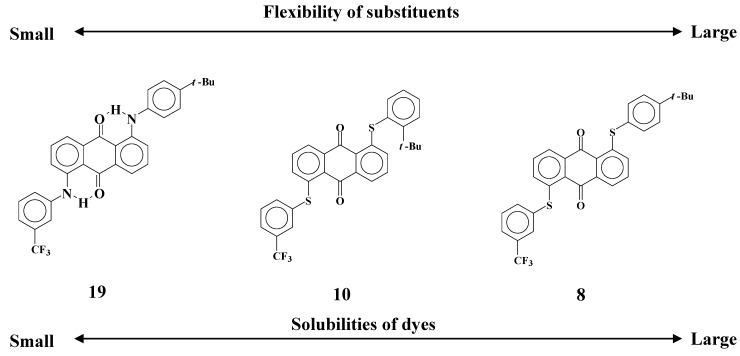
The relationships between flexibility of substituents and solubilities of dyes with analogous molecular structures.

Comparison of dyes **8**, **10** and **19** reveals that solubilities and dichroic ratios are closely related to the flexibility of the substituents as shown in Figure 15. 

**Figure 16:**
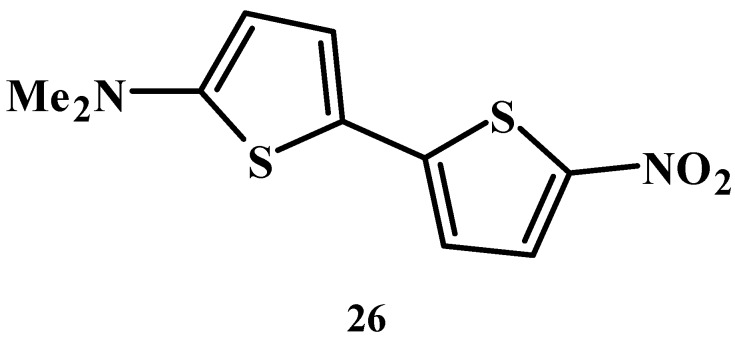
The molecular structure of donor-acceptor-substituted oligothiophene compound **26**.

In the liquid crystal phase, specific structures of solvation, which is different from the solvation in isotropic solutions, are formed and influence the solubility. And flexibility of substituents is very important for ensuring a suitable conformation matching the liquid crystal phase [29]. Donor-acceptor-substituted oligothiophene compound **26** shown in Figure 16 is known to show notable solvatochromic behaviors [31,32].

The solvatochromic behaviors, dielectric constants and solubilities of the dichroic dyes are thought to be correlated. Solvents with large dielectric constant behave as good ionic mediums. These solvents are considered effectively to stabilize the dyes in excited states, which have more polar structures than dyes in ground states. The stabilization of dyes in excited states induces longer wavelength shifts of the maximum absorption wavelengths of dyes; this is the origin of positive solvatochromic behaviors. On the other hand, solvents with large dielectric constants are considered to dissolve polar compounds such as dyes more effective than the solvents with small dielectric constant; thus solvatochromic behaviors can be correlated with the solubilities of the dyes.

The relationship between dipole moments and solubilities of the dyes is explained as follows. Since a dipole moment shows the value of the polarity of one molecule, a chemical compound such as a dye with a large polarity can be more easily dissolved as the dipole moment of a solvent becomes larger. Also, larger solvent dipole moments will stabilize dyes in their excited state more effectively. The stabilization of dyes in excited states induces the solvatochromic behaviors. Thus, a solvent with a larger dipole moment will dissolve a larger amount of dye, and it will increase the value of the solvatochromic behaviors.

In liquid crystals, maximum wavelengths of dyes shift to longer wavelength region compared with an isotropic solvent with close dipole moments and dielectric constant, indicating the formation of specific structures of solvation. To clarify this hypothesis, solvatochromic dye **26** was dissolved in fluorinated liquid crystals, and heated to the clearing point and maximum absorption wavelengths were observed continuously. Below the temperature of the clearing point, the maximum absorption wavelength hardly shifted. However, at temperatures above the clearing point, it shifted sharply to a shorter wavelength [32]. This phenomenon was not due to the thermodynamic effects but indicated the change of structures of solvation because it was observed in various liquid crystals with different clearing points. 

**Figure 17:**
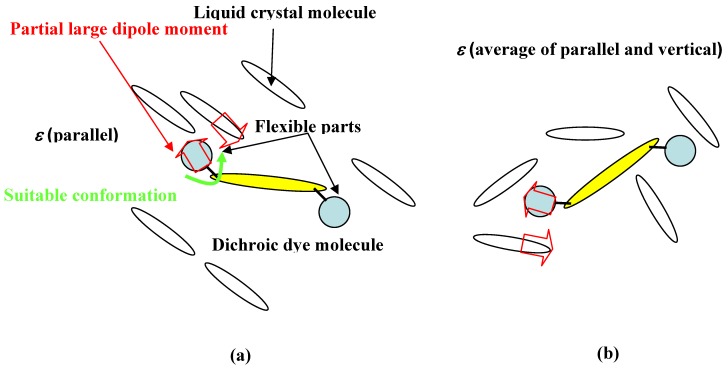
The model of structures of solvation. (a) in liquid crystal phase. (b) in isotropic phase.

In the liquid crystal phase, the dye aligns to the direction of the long axis of liquid crystal molecules and packing is closer than in the isotropic phase. With liquid crystals and dye molecules parallel to each other, strong dipole-dipole interactions between a polar part of flexible substituent with suitable conformation and rigid polar part of liquid crystals are realized. Moreover, in the liquid crystal phase, dye is influenced by the parallel dielectric constant which is larger than the vertical dielectric constant in the case of positive liquid crystals, inducing longer wavelength shift.

On the other hand, in the isotropic phase (with liquid crystals heated above their clearing point), the structures of solvation are converted to be analogous to those of a normal solvent. Dipole-dipole interaction between liquid crystals and dye molecules is weaker than in the liquid crystal phase, and dye is influenced by the average of parallel and vertical dielectric constant. Above all, the intensity of solvation is stronger in the liquid crystal phase than in the isotropic phase, and dyes with polar flexible substituents are highly soluble, forming a suitable conformation to adjust to the liquid crystal phase [29] (Figure 17).

## 3. Color Design and Adjustment of Novel Dichroic Dyes for Reflective Three-Layer GH-LCDs [33]

### 3.1. Constitutions of color simulations for selecting and adjusting dichroic dyes

Subtractive color mixing simulation was utilized to optimize the performances of the three-layer GH-LCDs [13]. The calculated spectra of three-layer GH-LCDs in yellow, magenta, cyan, green, blue, black and white states were converted into the CIE1976L^*^a^*^b^*^ color space. 

**Figure 18:**
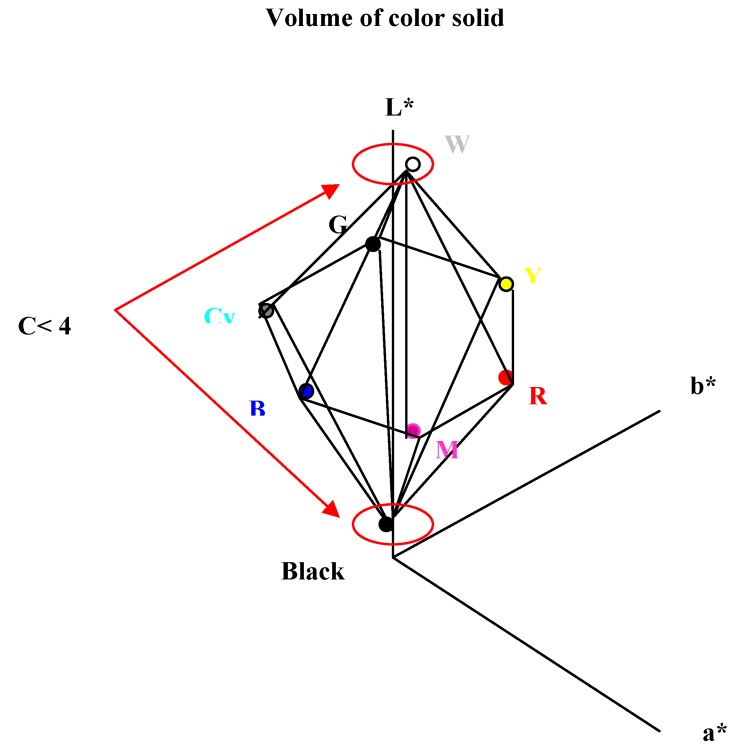
The color reproduction area of the three-layer GH-LCDs.

The light source was fixed as D65 and the CIE 1931 standard colorimetric system was used. The color reproduction area of the three-layer GH-LCD was defined as the volume inside the dodecahedron made by the above-mentioned eight color coordinates to simplify the calculation as shown in Figure 18. In fact, real color reproduction area is the volume inside the curved surface.

Maximizing the volume of the color solid is considered to be the best way to obtain excellent performances. Color balances in black and white states play a very important role in creating images with natural beauty. The restrictive condition such that the metric chromas of the achromatic colors [represented by equation (1)] were restricted below 4 was determined to adjust the color balance of the black and white states:

C* = ((a*)^2^ + (b*)^2^)^0.5^ < 4 (1)

### 3.2. The system of the three-layer GH-LCD and fundamental spectra data used in the simulation

The three-layer GH-LCD involves negative GH-liquid crystals with chiral compound that induce a helical structure in each layer. Both surfaces of each layer have alignment polymer that induces a homeotropic structure of liquid crystals. When voltage is applied to the cell, the helical structures are realized and dichroic dyes absorb a large quantity of light. In off states, liquid crystals are aligned vertically to the substrate and dichroic dyes absorb a small quantity of light. This is called a cholesteric-nematic phase transition system. Each layer is controlled independently and full color images are created. A cholesteric-nematic phase transition system using negative liquid crystals was selected to obtain large reflectance because of small anchoring energy and wide viewing angle.

The fundamental dye spectra used in the simulation were measured as follows. Dyes or dye mixtures were dissolved in negative fluorinated liquid crystals (ZLI-2806 purchased from Merck Ltd.). The chiral compound (S811, purchased from Merck Ltd.) was also dissolved in the liquid crystals. The concentration of chiral compound was determined such that the largest contrast was achieved with no hysteresis. GH-liquid crystals were injected in the cell and a voltage was applied. Spectra for both on and off states were measured. The spectra of off state can be calculated by active dichroic ratio (D’) defined by formula (2), and D’ can be determined freely in the program:

D’ = A (ON)/A(OFF)
(2)

where A(ON), A(OFF) are the absorbance at the maximum absorption wavelength of on and off states

### 3.3. Color adjustment of yellow and magenta layers from the viewpoint of absorption spectra

The effect of absorption spectra and dichroic ratios on the color reproduction areas (volume of color solid) can be individually investigated by subtractive color mixing simulation. At first, active dichroic ratio of three layers was set equal (D’=3.9: measured value of cyan dye) and absorption spectra were changed. Other calculation conditions were determined so that reflectance of white state was above 30%, that of black state was above 5%, and metric chroma C* was below 4. The largest volume of color solid was 38558, which was realized when coumarin dye **15** was used in yellow layer and anthraquinone dye **22** was used in magenta layer.

**Figure 19:**
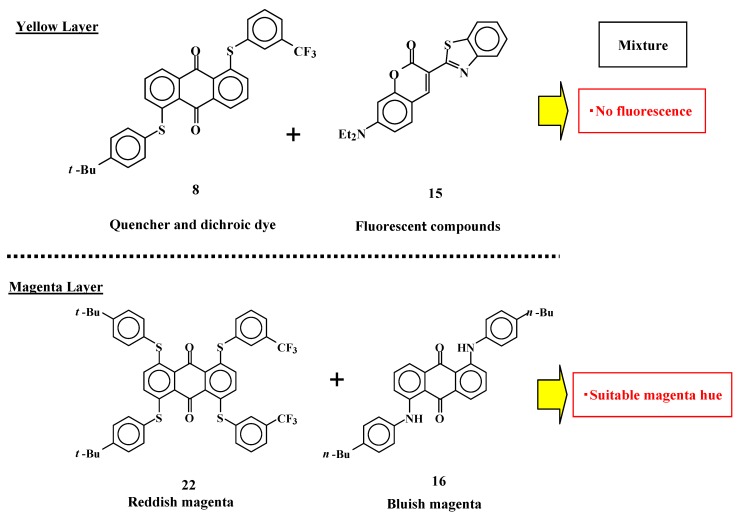
The dyes and/or dye mixtures for the three-layer GH-LCD of yellow and magenta layers.

However, coumarin dye **15** has a strong fluorescence that affects the hues of three-layer GH-LCD exhibits. The mixture of anthraquinone dye **8** and coumarin dye **15** is considered to be the best for realizing a large absorption coefficient, pure yellow color and no fluorescence because anthraquinone dyes with two phenylthio groups at the 1 and 5 positions behave as efficient fluorescence quenchers for coumarin dye (Figure 19) [30]. 

For the magenta layer, a mixture of reddish magenta dye **16** and bluish magenta dye **22** was selected from the viewpoint of color balance. SI-497 (purchased from Mitsui Chemicals Inc.) was used as cyan dye which gives a preferable result for color reproduction area.

Two combination of yellow, magenta and cyan dyes for three layered GH-LCDs were defined as follows: combination A (yellow: **6**, magenta:**16**, cyan: SI-497), combination B (yellow: **8+15**, magenta:**16+22**, cyan: SI-497). Combination A realize the largest volume of color solid only using commercially available dyes from the results of the calculation, and combination B realizes the largest volume of color solid using dyes suitable for the three-layer GH-LCDs obtained by our original dye technology shown in Figure 19.

**Figure 20:**
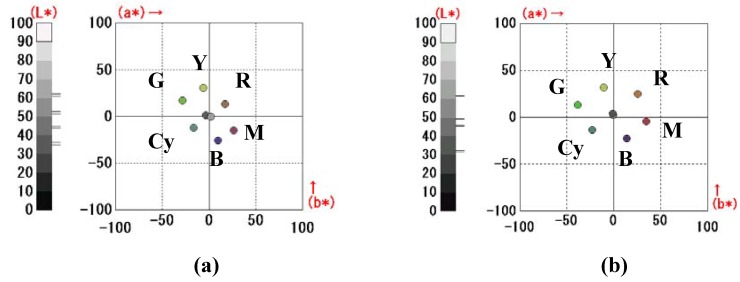
The calculated color coordinates of combinations A and B using measured D’ **(a)** Combination A, **(b)** Combination B.

As shown in Table 9, the volume of color solid calculated by measured D’ of combination B was 1.6 times larger than that of combination A due to the effects of improved absorption spectra. The calculated color coordinates of combinations A and B using measured D’ are shown in Figure 20. Marked improvement of color reproduction area is observed for combination B compared with combination A. In particular, the chroma of red is drastically increased.

**Table 9:** The dye combination and calculated volumes of color solid (measured D’ of combination A and B were used in calculations).

Dye combination	Yellow	Magenta	Cyan	Calculated volume of color solid
A	6	16	SI-497	17423
B	8+15	22+16	SI-497	27326

**Figure 21:**
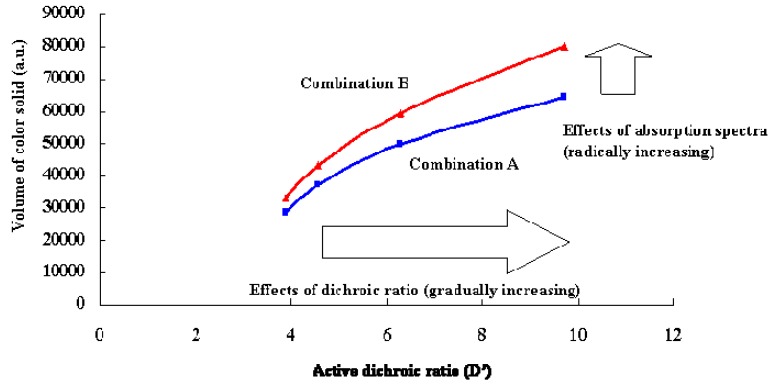
The calculated effects of active dichroic ratios on the volume of color solids.

### 3.4. Effects of active dichroic ratios of dyes on color reproduction areas.

To ascertain the effects of active dichroic ratios (D’) of dyes on the volume of color solid, dichroic ratios of dyes and or dye mixtures of combinations A and B were changed by simulation. Calculated results of volume of color solid when D’ was a variable are shown in Figure 21. By increasing dichroic ratios, volumes of color solids are enlarged monotonously in both combinations. However, differential coefficient of combination B is considerably larger than that of combination A. This indicates absorption spectra are key factors for accelerate the effects of enlarged dichroic ratios.

### 3.5. The prototype of the reflective three-layer GH-LCD and its performances

**Figure 22:**
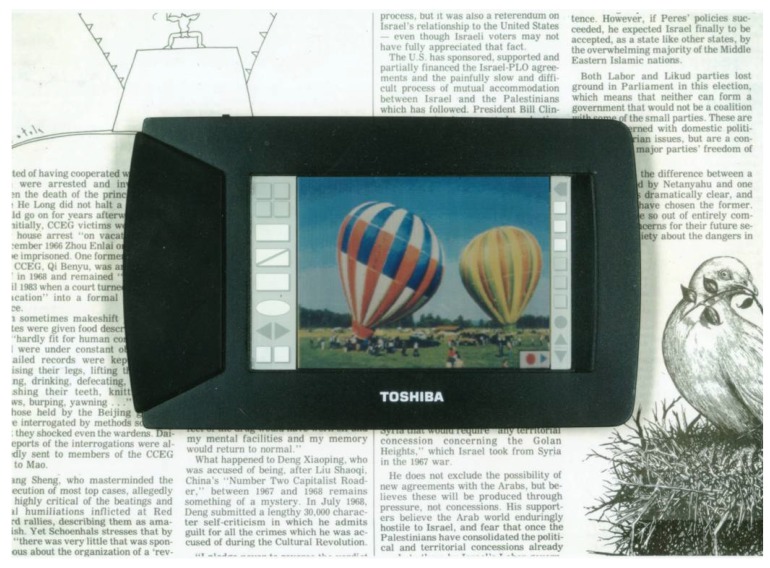
The color image of the reflective three-layer GH-LCD (the LCD is on a newspaper. Large reflectance in white state (43 %) is demonstrated.) [33].

A prototype of the reflective three-layer GH-LCD using dyes and/or dye mixtures of combination B was produced. A color image is shown in Figure 22 and performances are listed in Table 10. A reflective three-layer GH-LCD with pure colors and bright image was created.

**Table 10:** The performances of the prototype of the reflective three-layer GH-LCD [33].

**Size of Panel**	3.4 inches (diagonal)
**Number of Pixels**	240(H) 160(V)
**Size of Pixels**	0.3 mm 0.3 mm
**Contrast**	5.3
**Reflectance in white state**	43%

## 4. Conclusions

Novel yellow and magenta anthraquinone dyes that are highly soluble in fluorinated liquid crystals even at low temperatures and have large dichroic ratios were developed. These highly soluble dyes with flexible substituents form suitable conformations in liquid crystals to adjust to the liquid crystal phase. A prototype of the three-layer GH-LCD using highly soluble yellow and magenta dichroic dyes and dye mixtures was made and their performances were investigated. The color reproduction area was 1.6 times larger than that of the best combination of commercially available dichroic dyes and pure colors and bright image were created.

## Supplementary Materials

Supplementary File 1
