# Iwanaga, H. Development of Highly Soluble Anthraquinone Dichroic Dyes and Their Application to Three-Layer Guest-Host Liquid Crystal Displays. *Materials* 2009, *2*, 1636-1661

**DOI:** 10.3390/ma4010103

**Published:** 2010-12-30

**Authors:** Hiroki Iwanaga

**Affiliations:** Corporate Research & Development Center, Toshiba Corporation, 1 Komukai-Toshiba-cho, Saiwai-ku, Kawasaki 212-8582, Japan; E-Mail: hiroki.iwanaga@toshiba.co.jp; Tel.: +81-44-549-2174; Fax: +81-44-549-2387

I found some mistakes on Table 2 (page 1643) in my published paper in *Materials* [1]. A correct table is provided here.

**Table 2:** The relationships between molecular structures of yellow anthraquinone dyes and their solubilities and dichroic ratios.

Dye	Solubility (wt %) ^1)^		Dichroic ratio
	r. t.	Low temp. ^2)^	
6	4	1.7	10
7	3.1	2.1	11
8	5.8	3.5	10
9	0.66	0.38	10
^1)^ Measured in fluorinated liquid crystals (LIXON 5052 XX)			
^2)^ 267-268 K			
