# Supplementary material for: Development of Highly Soluble Anthraquinone Dichroic Dyes and Their Application to Three-Layer Guest-Host Liquid Crystal Displays
Source: Materials (Basel). 2009 Oct 23;2(4):1636–61. doi: 10.3390/ma2041636 (PMC5513388; doi:10.3390/ma2041636)
Supplement: Supplementary File 1 [file materials-02-01636-s001.pdf]

Materials **2011**, *4*

OPEN ACCESS

materials

ISSN 1996-1944

www.mdpi.com/journal/materials

Correction

Iwanaga, H. Development of Highly Soluble Anthraquinone Dichroic Dyes and Their Application to Three-Layer Guest-Host Liquid Crystal Displays. *Materials* 2009, *2*, 1636-1661

Hiroki Iwanaga

Corporate Research & Development Center, Toshiba Corporation, 1 Komukai-Toshiba-cho, Saiwai-ku, Kawasaki 212-8582, Japan; E-Mail: hiroki.iwanaga@toshiba.co.jp; Tel.: +81-44-549-2174; Fax: +81-44-549-2387

Received: 14 December 2010 / Accepted: 23 December 2010 / Published: 30 December 2010

I found some mistakes on Table 2 (page 1643) in my published paper in *Materials* [1]. A correct table is provided here.

Table 2. The relationships between molecular structures of yellow anthraquinone dyes and their solubilities and dichroic ratios.

Dye	Solubility (wt %) [1)]		Dichroic ratio
	r. t.	Low temp. [2)]	
6	4	1.7	10
7	3.1	2.1	11
8	5.8	3.5	10
9	0.66	0.38	10

[1)] Measured in fluorinated liquid crystals (LIXON 5052 XX)

[2)] 267-268 K

References

1. Iwanaga, H. Development of Highly Soluble Anthraquinone Dichroic Dyes and Their Application to Three-Layer Guest-Host Liquid Crystal Displays. *Materials* **2009**, *2*, 1636-1661.

© 2010 by the authors; licensee MDPI, Basel, Switzerland. This article is an open access article distributed under the terms and conditions of the Creative Commons Attribution license (http://creativecommons.org/licenses/by/3.0/).
